# Changes in urethral smooth muscle and external urethral sphincter function with age in rats

**DOI:** 10.14814/phy2.14643

**Published:** 2020-12-23

**Authors:** Takuma Oshiro, Ryu Kimura, Keiichiro Izumi, Asuka Ashikari, Seiichi Saito, Minoru Miyazato

**Affiliations:** ^1^ Department of Urology Graduate School of Medicine University of the Ryukyus Okinawa Japan; ^2^ Department of Systems Physiology Graduate School of Medicine University of the Ryukyus Okinawa Japan

**Keywords:** aging, detrusor underactivity, synergy

## Abstract

To confirm changes in urethral activity with age, both intravesical pressure and urethral perfusion pressure (UPP) were recorded and external urethral sphincter electromyography (EUS‐EMG) was performed. A total of 33 female Sprague Dawley rats aged 3 months (young rats), 12 months (middle‐aged rats), and 24 months (aged rats) were used. Bladder activity was evaluated using continuous cystometry. Urethral activity was evaluated by simultaneously recording intravesical pressure and UPP in isovolumetric conditions under urethane anesthesia in each group. Additionally, EUS‐EMG activity was monitored under the same conditions. In continuous cystometry, the amplitude of bladder contractions was not different among the three groups; nevertheless, residual urine volume was significantly increased in middle‐aged and aged rats, as compared in young rats. With respect to UPP, the change in UPP was significantly smaller in aged rats (60%) and middle‐aged rats (64%) than in young rats. Furthermore, the mean amplitude of high‐frequency oscillations of the EUS was significantly lower in aged (61%) and middle‐aged rats (70%) than in young rats. EUS‐EMG revealed EUS bursting activity during voiding with clear active and silent phases in young rats but unclear active and silent phases in aged rats. Masson's trichrome staining of the urethra showed EUS atrophy in aged rats compared to young and middle‐aged rats. The results indicate that aging induces two urethral dysfunctions in the urethral smooth muscle and EUS, which may lead to dyscoordination between the urinary bladder and urethra.

## Graphical abstract

To confirm changes in urethral activity with age, both intravesical pressure and urethral perfusion pressure (UPP) were recorded and external urethral sphincter electromyography (EUS‐EMG) was performed. The amplitude of bladder contractions was not different among young, middle‐aged, and aged rats; nevertheless, residual urine volume was significantly increased in middle‐aged and aged rats and the change in UPP was significantly smaller in aged rats. In histological findings, EUS hypotrophy was observed in aged rats. The results indicate that aging induces two urethral dysfunctions in the urethral smooth muscle and EUS, which may lead to dyscoordination between the urinary bladder and urethra.

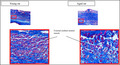

## 1 INTRODUCTION

Aging leads to complicated changes in bladder activity, including detrusor hyperactivity and/or detrusor underactivity (DU) (Abrams et al., 2003; Jeong et al., 2012). Furthermore, aging is associated with lower urinary tract symptoms, resulting in increased health problems (Jeong et al., 2012). The aging bladder is thought to be induced by multifactorial pathogenesis, such as impaired endothelial dysfunction largely mediated by nitric oxide (NO) insufficiency via oxidative stress, chronic low‐grade inflammation, and loss of nerve fibers (Abrams et al., 2003; Bachschmid et al., 2013; Jeong et al., 2012; Yamamoto et al., 2006). In our previous study, middle‐aged (i.e., 12 month old) rats exhibited decreased bladder contractions and increased residual urine volume (i.e., DU) under urethane anesthesia (Oshiro et al., 2014). Micturition is a physiological phenomenon that requires coordination between the bladder and urethra (de Groat & Yoshimura, 2001). However, little is known about age‐associated changes in urethral function when compared to aging bladder dysfunction.

Acetylcholine is released from parasympathetic nerves to contract the bladder, whereas NO is released into the urethra to relax the urethral smooth muscle (Maggi et al., 1986; Miyazato et al., 2008). The striated muscles of the external urethral sphincter (EUS) also relax during voiding. EUS‐electromyography (EMG) recordings in normal rats show tonic activity prior to the onset of voiding and bursting activity during voiding. This bursting activity is speculated to generate high‐frequency oscillations (HFOs) measured as urethral perfusion pressure (UPP) induced by rhythmic contractions and EUS relaxation, which contribute to urethral pumping activity in rats and dogs. The pumping activity of the EUS does not occur in humans (Maggi et al., 1986; Miyazato et al., 2008; Nishizawa et al., 1984). In our recent study, middle‐aged (i.e., 12–15 month old) rats exhibited an impairment in urethral smooth muscle relaxation and a reduction in the HFOs of the EUS (Kimura et al., 2018). Nonetheless, age‐related changes in two different urethral mechanisms and the relationship between them have not been understood.

Therefore, the present study aimed to investigate time‐course changes in bladder and urethral functions in young, middle‐aged, and aged rats using cystometrography with conscious restraint, as well as simultaneous recordings of intravesical pressure and UPP with EUS‐EMG. Additionally, Masson's trichrome staining was performed to confirm histologic changes in the urethral structure with age.

## 2 MATERIALS AND METHODS

### 2.1 Animals

A total of 33 female Sprague Dawley rats fed with a standard diet was categorized into young rats (age: 3 months; *n* = 15), middle‐aged rats (age: 12 months; *n* = 9), and aged rats (age: 24 months; *n* = 13). The study protocol was submitted to and approved by the animal ethics committee of the University of the Ryukyus; furthermore, the protocol was performed under a license obtained from this committee.

### 2.2 Experiment 1: Recordings of intravesical pressure (continuous cystometry) under awake conditions

Continuous cystometry under awake conditions was conducted on young (*n* = 8), middle‐aged (*n* = 8), and aged (*n* = 8) rats. To perform cystometry, the bladder and ureters were exposed via a lower midline abdominal incision. The bilateral ureters were cut, the distal ends were tied, and a polyethylene catheter (PE‐90, Intramedic; Clay Adams) was inserted into the bladder through the bladder dome. Following the closure of the abdomen, the rats were placed in a restrainer (Braintree Scientific Inc.) and were allowed to recover from anesthesia for 1–2 hr. Bladder activity was monitored via a catheter connected to a pressure transducer and a saline infusion pump through a three‐way stopcock. PowerLab (ADInstruments Pty. Ltd.) was used for data acquisition and manipulation. To induce rhythmic contractions, the bladder was infused with physiological saline at a rate of 0.05 ml/min. Cystometry was continued for at least 60 min. The intercontraction interval (ICI), pressure threshold, baseline pressure, amplitude voiding pressure, and the number of non‐voiding contractions (NVCs) were measured during the final 30 min. NVCs were defined as small‐amplitude bladder contractions without voiding that were greater than 3 cmH_2_O (Choo et al., 2018). During the final bladder contraction, bladder infusion was stopped and the residual urine volume was measured by withdrawing intravesical fluid through the catheter by gravity and compressing the bladder.

### 2.3 Experiment 2: Recordings of isovolumetric intravesical pressure, UPP, and EUS‐EMG under urethane anesthesia

**FIGURE 1:**
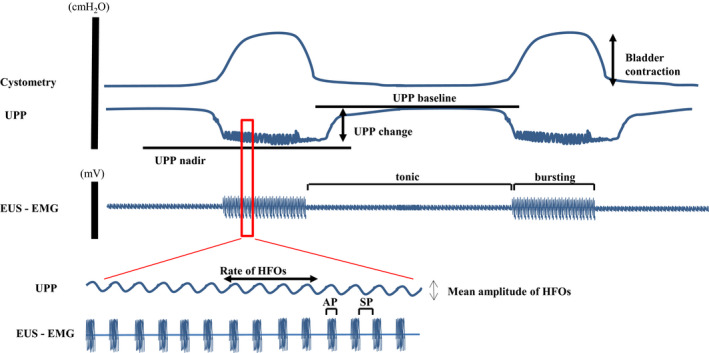
Simultaneous recording of triple parameters of intravesical pressure, UPP, and EUS‐EMG under isovolumetric conditions. EUS‐EMG, external urethral sphincter‐electromyography; HFOs, high‐frequency oscillations; UPP, urethral perfusion pressure

Isovolumetric cystometry and recordings of UPP and EUS‐EMG were performed in young (*n* = 5), middle‐aged (*n* = 5), and aged (*n* = 3) rats. The rats were anesthetized with isoflurane, and a polyethylene catheter (PE‐10) was inserted into the jugular vein for drug injection. A polyethylene catheter (PE‐90) was also inserted into the bladder, as in experiment 1, with the bilateral ureters cut. The bladder neck was tied below the vesicoureteral junction to prevent injury to the pelvic nerve, in which bladder and urethral activities can be measured separately. Following the closure of the abdomen, isoflurane anesthesia was turned off and replaced with urethane anesthesia (0.8–1.2 g/kg). A PE‐50 was also inserted into the urethra from the external urethral orifice. EUS‐EMG activity was recorded using two 500‐µm polyurethane‐coated stainless needle electrodes (Bio Research Center Co., Ltd.) inserted into both sides of the EUS under direct visualization. These were connected to a computer where the cystometrogram and EUS‐EMG data were amplified and digitized using a computer system equipped with an analog‐to‐digital converter (PowerLab). To induce rhythmic isovolumetric contractions, the bladder was infused with physiological saline at a rate of 0.05 ml/min until rhythmic contractions were confirmed. The urethral catheter was continuously infused with physiological saline at a rate of 0.05 ml/min. When the rhythmic bladder contractions stabilized for at least 30 min, the maximum bladder contractions and intravesical pressure threshold for inducing urethral relaxation were measured. The amplitude of voiding bladder contractions was defined as the maximum bladder contractions minus the baseline pressure. Urethral pressure was altered by bladder contractions. Thus, certain parameters of UPP during bladder contractions—namely, UPP nadir during reflex urethral relaxation and baseline UPP between reflex bladder contractions—were measured. The change in UPP was calculated as the UPP nadir minus the baseline UPP. Additionally, the mean rate and amplitude of HFOs of the urethral striated muscles during reflex bladder contractions were measured (Figure 1). These parameters were averaged over 30 min and compared among the three groups. EUS‐EMG activity was also evaluated by the ratio of the active phase (AP) (s) and silent phase (SP) (s) during bladder contractions (AP/ [AP + SP]). Finally, α‐bungarotoxin (225 µg/kg, Sigma‐Aldrich Co.), a neuromuscular nicotinic receptor antagonist, was intravenously injected into two rats in each group to suppress EUS activity. After that, the respiratory condition was carefully observed and oxygen was administered.

### 2.4 Experiment 3: Masson's trichrome staining

To confirm histological changes in the bladder with age, the urethra was removed from the remaining young rats at 3 months (*n* = 2) and aged rats at 24 months (*n* = 2). Masson's trichrome staining was performed according to a standard protocol. The harvested urethras were fixed in 10% neutral buffered formalin, and 5‐μm‐thick sections were cut from paraffin‐embedded specimens on a microtome and thawed onto gelatin‐coated slides. Following deparaffinization in xylene and ethanol, tissue sections were stained with Hansen's iron hematoxylin for 5 min. After the sections were washed with running tap water for 5 min, they were stained with Biebrich scarlet‐acid fuchsin for 10 min. After the sections were further rinsed in distilled water, they were treated with phosphomolybdic acid for 10 min. Subsequently, the sections were stained with light green instead of methylene blue for 10 min to avoid any possible confusion between blue nuclear staining and collagen staining. In Masson's trichrome staining, the stained components were identified as follows: nuclei were stained black or blue; cytoplasm, muscle, and erythrocytes were stained red; and collagen fibers were stained green. Next, the tissue sections were dehydrated in a graded ethanol series, cleared in xylene, mounted with Permount, (FALMA Co., Ltd.), and placed under coverslips.

### 2.5 Statistical analysis

Results are presented as mean ± *SE*. Between‐group comparisons were performed using the Mann–Whitney *U*‐test. *p* < .05 was considered statistically significant.

## 3 RESULTS

### 3.1 Comparison of body weight among the three groups

The body weight was significantly higher in middle‐aged and aged rats (554 ± 47.6 g and 693.7 ± 39.5 g, respectively; *p* < .01) than in young rats (254.7 ± 6.7 g).

### 3.2 Differences in intravesical pressure and residual urine volume under awake conditions among the three groups (experiment 1)

**FIGURE 2:**
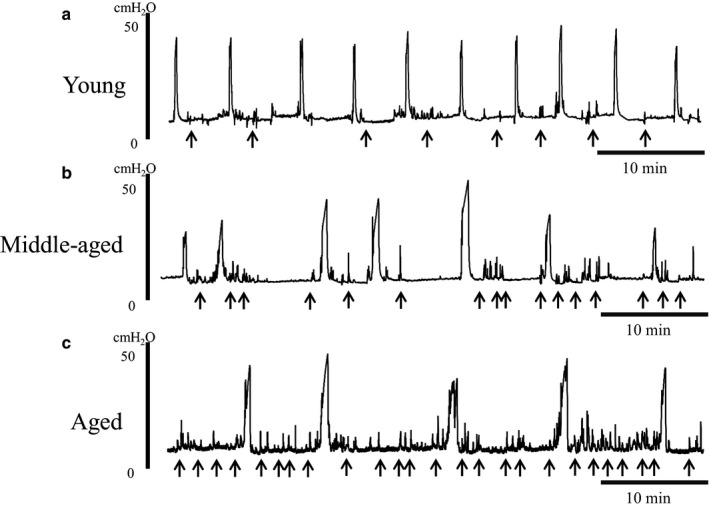
Recording of intravesical pressure under awake conditions. Arrows indicate non‐voiding contractions (NVCs). The amplitude of voiding bladder contractions was not significantly different among young, middle‐aged, and aged rats. However, the number of NVCs was significantly increased in aged rats

Under awake conditions, the amplitude of voiding bladder contractions was not significantly different among young, middle‐aged, and aged rats (34.9 ± 5.5 vs. 25.9 ± 1.0 vs. 29.3 ± 1.9 cmH_2_O). However, the residual urine volume was significantly increased in middle‐aged and aged rats (0.18 ± 0.04 and 0.36 ± 0.18 ml, respectively; *p* < .05) when compared to that in young rats (0.00 ± 0.00 ml). The bladder contraction time was significantly increased in middle‐aged and aged rats (20.3 and 18.3 s, respectively; *p* < .01), as compared to that in young rats (11.1 s). The baseline intravesical pressure and intravesical pressure threshold for inducing bladder contraction were not significantly different among the three groups. The ICI was significantly prolonged in middle‐aged and aged rats (700 and 973 s, respectively; *p* < .01) when compared to that in young rats (197 s). The number of NVCs was significantly increased in aged rats (Table 1, Figure 2).

**TABLE 1:** Intravesical parameters under awake conditions among young, middle‐aged, and aged rats

	Young (*n* = 8) mean ± SE	Middle aged (*n* = 8) mean ± SE	Aged (*n* = 8) mean ± SE
Base line pressure (cmH_2_O)	8.24 ± 2.53	7.88 ± 0.08	6.15 ± 1.32
Threshold pressure (cmH_2_O)	11.91 ± 2.78	11.25 ± 0.31	11.36 ± 2.53
Amplitude of bladder contraction (cmH_2_O)	34.94 ± 5.48	25.91 ± 1.04	29.34 ± 1.91
Contraction time (s)	11.1 ± 1.68	20.31 ± 1.73**	18.31 ± 1.44**
ICI (s)	197.36 ± 36.27	700.00 ± 83.85**	973.65 ± 170.03**
NVCs (Hz)	3.63 ± 0.3	4.5 ± 0.90	7.50 ± 0.56**, †
Residual urine volume (ml)	0.00 ± 0.00	0.18 ± 0.04*	0.38 ± 0.03*

Note

Values are presented as mean ± standard error (SE).

Abbreviations: ICI, intercontraction intervals; NVCs, non‐voiding contractions

* *p* < .05 versus young rats,

** *p* < .01 versus young rats,

^†^ *p* < .05 versus middle aged rats

### 3.3 Differences in intravesical pressure, UPP, and EUS‐EMG under urethane anesthesia among the three groups (experiment 2)

**FIGURE 3:**
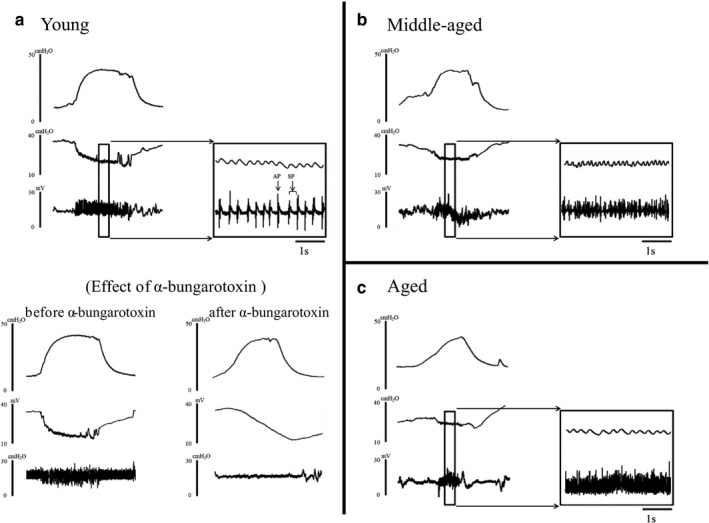
Representative traces of simultaneous recording of intravesical pressure, UPP, and EUS‐EMG during isovolumetric contractions. (a) During EUS bursting with urethral relaxation, the active phase (AP) and silent phase (SP) were detected in young rats. After an α‐bungarotoxin injection, the HFOs of UPP and EUS‐EMG bursting activity disappeared. Furthermore, the negative shift in UPP during bladder contractions was enlarged. (b) In middle‐aged rats, the AP tended to be extended and the border of the AP and SP became unclear. (c) In aged rats, the border of AP and SP could not be distinguished. UPP, urethral perfusion pressure; EUS‐EMG, external urethral sphincter‐electromyography

In experiment 1, as the residual urine volume increased with age without affecting bladder contractions, simultaneous recordings of intravesical pressure and UPP were performed to confirm changes in urethral activity with age. Under isovolumetric conditions, UPP measurements indicated urethral relaxation accompanied by HFOs of the urethral striated muscles during reflex bladder contractions in young, middle‐aged, and aged rats (Figure 3). Furthermore, the amplitudes of bladder contractions were significantly different among the groups (Table 2). Baseline UPP was not significantly different among the three groups. The mean UPP nadir during large‐amplitude bladder contractions was significantly different between young and aged rats (22.5 ± 6.1 vs. 29.5 ± 7.6 cmH_2_O; *p* < .05) but was not significantly different between young and middle‐aged rats. However, the change in UPP during urethral relaxation was significantly smaller in middle‐aged and aged rats (−9.79 ± 6.9, −9.2 ± 8.6, respectively, *p* < .05) than in young rats (−15.3 ± 5.5 cmH_2_O).|UPP change|/bladder contraction was also significantly lower in middle‐aged rats than in young rats (0.33 ± 0.02 vs. 0.24 ± 0.02; *p* < .01); nevertheless, no significant change was noted in aged rats (0.30 ± 0.02; *p* = .50). The amplitude of HFOs was significantly decreased in middle‐aged and aged rats (2.9 ± 1.9 and 2.5 ± 1.3 cmH_2_O, respectively; *p* < .05) when compared to that in young rats (4.1 ± 2.6 cmH_2_O).

**TABLE 2:** Changes in the parameters of bladder and urethral functions among young, middle‐aged, and aged rats

	Young (*n* = 5) mean ± SE	Middle aged (*n* = 5) mean ± SE	Aged (*n* = 3) mean ± SE
Amplitude of bladder contraction (cmH_2_O)	47.9 ± 7.8	42.0 ± 8.6*	30.2 ± 10.2*
UPP baseline (cmH_2_O)	37.3 ± 5.6	34.8 ± 10.1	38.2 ± 6.2
UPP nadir (cmH_2_O)	22.5 ± 6.1	25.6 ± 12.3	29.5 ± 7.6*
UPP change (cmH_2_O)	−15.3 ± 5.5	−9.79 ± 6.9*	−9.2 ± 8.6*
HFOs amplitude (cmH_2_O)	4.1 ± 2.6	2.9 ± 1.9*	2.5 ± 1.3*
Rate of HFOs (Hz)	3.8 ± 0.3	4.3 ± 0.3	3.5 ± 0.4
AP (s)	0.055 ± 0.002	0.037 ± 0.002	—
SP (s)	0.13 ± 0.007	0.067 ± 0.004	—
AP/ (AP + SP)	0.28 ± 0.014	0.354 ± 0.022 *	—

Note

Values are presented as mean ± standard error (SE).

Abbreviations: AP, active phase; HFOs, high‐frequency oscillations; SP, silent phase; UPP, urethral perfusion pressure.

* *p* < .05 versus young rats.

EUS‐EMG indicated bursting in young, middle‐aged, and aged rats during bladder contractions. The AP during bladder contractions coincided with the HFOs of UPP. In young rats, EUS‐EMG revealed EUS bursting activity during bladder contractions with clear APs and SPs. In middle‐aged rats, the APs were extended and the border of APs and SPs was unclear. The time ratio of EUS‐EMG bursting (AP/ [AP + SP]) was significantly greater in middle‐aged rats than in young rats (0.358 ± 0.025 vs. 0.280 ± 0.014; *p* < .05). In aged rats, EUS‐EMG showed bursting with a long duration of APs. Due to the long duration of APs, SPs were not observed and could not be calculated (AP/ [AP + SP]). (Table 2, Figure 3).

After α‐bungarotoxin injection, the HFOs of UPP and EUS‐EMG bursting activity completely disappeared in all groups. Furthermore, the negative shift in UPP during bladder contractions was enlarged (Figure 3).

### 3.4 Differences in urethral histological findings between young and aged rats by Masson's trichrome staining

**FIGURE 4:**
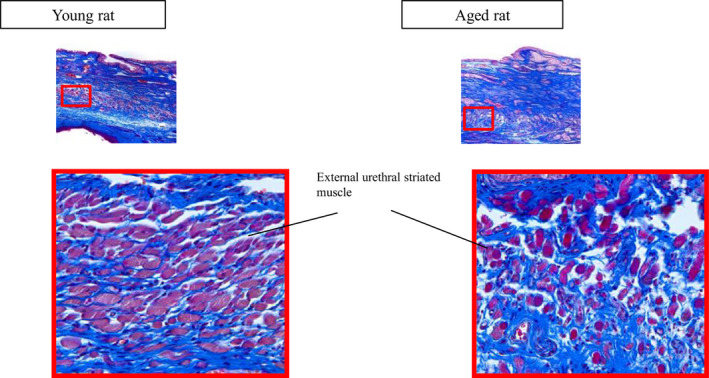
Masson's trichrome staining of the urethra. EUS atrophy and fibrous tissue hyperplasia are observed in the submucosal layer in aged rats compared with young rats. EUS, external urethral sphincter

Masson's trichrome staining revealed EUS atrophy in the urethra of aged rats, as compared to that of young rats (Figure 4). Additionally, there were more fibrous tissues in the submucosal layer of the urethra in aged rats than in young rats.

## 4 DISCUSSION

The present study yielded several important findings. First, continuous cystometry under awake conditions showed that the residual urine volume was significantly increased in middle‐aged and aged rats, as compared to that in young rats. However, the amplitude of bladder contractions was not significantly different among the three groups, suggesting changes in the urethral function with age. Therefore, as the next step, we examined the time‐course changes in UPP using EUS‐EMG under isovolumetric bladder conditions and urethane anesthesia. Second, similar to our recent data (Kimura et al., 2018), the change in UPP during bladder contractions was significantly decreased in both middle‐aged and aged rats when compared to that in young rats, suggesting impairment in urethral smooth muscle relaxation with age. Third, EUS‐EMG revealed bursting activity during bladder contractions with a clear AP and SP in young rats but an unclear AP and SP in aged rats, suggesting impairment in EUS activity with age. Thus, it is reasonable to assume that aging induces two urethral dysfunctions in the urethral smooth muscle and EUS during voiding.

EUS relaxation is an important factor for voiding efficiency and contributes to the reduction in residual urine volume. HFOs are prompted by the rhythmic contractions of the EUS, contributing to urethral pumping activity in rats and dogs (Maggi et al., 1986; Miyazato et al., 2008; Nishizawa et al., 1984). Thus, it is thought to be part of the coordinated urethral activity during bladder contractions. Similar to our recent study (Kimura et al., 2018), the amplitude of HFOs during urethral relaxation was significantly lower in middle‐aged and aged rats than in young rats in this study. Our novel study on the triple recording of EUS‐EMG also showed bursting activity during bladder contractions with a clear AP and SP in young rats, an unclear AP and SP in middle‐aged rats, and no SP in aged rats. The AP during bladder contractions coincided with the HFOs of UPP, whereas the SP was the relaxation of HFOs like a run‐up period. The disappearance of SP (and longer duration of AP) in aged rats may suggest tonic and detrusor‐sphincter dyssynergia‐ like changes even during the UPP negative shift (i.e., synergic). This strange change mimics urethral contraction during UPP relaxation in streptozotocin‐induced diabetic rats (Torimoto et al., 2004). Following the injection of α‐bungarotoxin, a neuromuscular blocker, the HFOs of UPP and EUS‐EMG bursting activity completely disappeared, and the negative shift in UPP during bladder contractions was enlarged. It has been reported that a positive shift in UPP during bladder contractions (i.e., detrusor‐sphincter dyssynergia) was changed to synergic after inhibition of EUS activity by intrathecal γ‐aminobutyric acid agonist in rats with transected spinal cords (Miyazato et al., 2008). Therefore, it is assumed that the negative shift in UPP may originate from the sum of EUS and urethral smooth muscle relaxation. In the present study, Masson's trichrome staining revealed EUS atrophy in the urethra of aged rats, as compared to that of young rats. There were also more fibrous tissues in the submucosal layer of the urethra in aged rats than in young rats. Human studies reported that the number and density of urethral striated muscle fibers removed from 25 cadaveric females aged 15–80 years declined with age (Perucchini et al., 2002; Perucchini et al., 2002). Thus, EUS activity may be impaired as a consequence of the decreasing density of the EUS with age. EUS bursting activity and pressure oscillations in cystometrograms are abolished by α‐bungarotoxin or pudendal nerve transection in rats (Conte et al., 1991; Maggi et al., 1986; Peng et al., 2008; Yoshiyama et al., 2000). In the study conducted by Morita et al. (Morita et al., 1984), the EUS exhibited a normal synergic electromyogram pattern in 7 out of 15 dogs even after bilateral pudendal nerve transection; however, it disappeared after bilateral pelvic nerve transection. They also reported that horseradish peroxidase‐positive cells were found in Onuf's nucleus of the sacral cord in half of the dogs whose pelvic nerves were injected with this dye. These results suggest that the pelvic nerve may also contain somatic fibers innervating the EUS and facilitate HFOs. Overall, EUS pumping and relaxation play an important role in the coordination between the bladder and urethra, and may be impaired anatomically or neurogenically with age.

Urethral smooth muscle relaxation is crucial for efficient voiding. Similar to the findings of our recent study (Kimura et al., 2018), the change in UPP during bladder contractions was significantly decreased in middle‐aged (i.e., 12‐month) rats, as compared to that in young rats in this study. We also confirmed the decreased UPP change in aged rats (24 months) and obtained the same results. In our recent study, urethral contraction during urethral relaxation was noted in middle‐aged rats, which disappeared after treatment with Arg, an NO substrate (Kimura et al., 2018). Alexandre et al. (Alexandre et al., 2014) reported that NO donor‐mediated urethral smooth muscle relaxation was impaired in obese mice, and this impairment could be reversed by BAY 60–2770, a soluble guanylyl cyclase activator. These results suggest that numerous general age‐associated changes, such as ischemia and inflammation (Rea et al., 2018), affect NO synthesis in the lower urinary tract as well, leading to dysfunctional urethral smooth muscle relaxation.

Furthermore, the present study showed that middle‐aged and aged rats had prolonged ICI, increased residual urine volume, and numerous NVCs without affecting bladder contractions in continuous cystometry under awake conditions. The bladder contraction time was also prolonged in middle‐aged and aged rats, as compared to that in young rats. Thus, aging is assumed to induce detrusor hyperactivity and/or DU, possibly due to the excitability of C‐fiber bladder afferents (Cheng et al., 1995). The present study identified no significant differences in the amplitude of bladder contractions with aging under awake conditions. However, under urethane anesthesia, the amplitude of bladder contractions was significantly lower with aging. Urethane is a widely used anesthetic in animal studies on the lower urinary tract; nonetheless, it is well known that it negatively affects cystometric pressure, micturition volume, and residual urine volume (Armstrong et al., 1982; Yoshiyama et al., 2013). Thus, it is suggested that urethane anesthesia may have a negative effect on the aging bladder. In contrast, changes in urethral activity depend on bladder activity; hence, the ratio of |UPP change| and bladder contraction under urethane anesthesia was also examined. |UPP change|/bladder contraction was also significantly lower in middle‐aged rats than in young rats; nevertheless, no significant change was noted in aged rats. These results suggest that changes in the urethral function predominantly occur in middle‐aged rats, whereas changes occur equally in both bladder and urethral functions in aged rats. Thus, with age, urethral dysfunction may occur earlier than bladder dysfunction. Overall, bladder function overlaps urethral dysfunction with age, which may lead to DU with a vicious cycle.

Age‐associated changes in the lower urinary tract induce detrusor hyperactivity with impaired bladder contractility (Abrams et al., 2003; Jeong et al., 2012). Thus, age‐associated urethral dysfunctions may lead to inefficient voiding with increased post‐void residual urine volume, which is often observed in elderly populations. As rats live an average of 2–3.5 years, one day of a rat's life is equivalent to approximately 35 human days. Therefore, the age categories used in this study are appropriate (Sengupta, 2013). The results of our present study imply that urethral dysfunction with age is also an important factor that may aggravate bladder dysfunction. The findings of this study may help elucidate the mechanisms involved in the genesis of dysfunctional aging bladders.

This study has several limitations. First, the study on EUS‐EMG with aging was performed under urethane anesthesia. Urethane activates sympathetic nerves by inducing an increase in serum epinephrine and norepinephrine levels (Armstrong et al., 1982). Additionally, how much influence urethane anesthesia has on the bladder and urethral functions with aging is not well understood. Second, we did not record the central or peripheral nervous system involved in the changes in EUS activity with aging. Despite these limitations, the results of the present study may be of significance for future studies on urinary dysfunction with aging.

In conclusion, the EUS contracts together with the urethral smooth muscle during the storage phase and relaxes during the voiding phase. Interestingly, the EUS can be under voluntary control via the pudendal nerve. This triple recording of bladder, UPP, and EUS‐EMG provides new evidence that the voluntary activity of the EUS worsens with aging. Our results suggest that EUS dysfunction accompanied by impairment in NO‐mediated urethral smooth muscle relaxation is one factor contributing to the development of an aging bladder with residual urine volume, urgency, or incontinence.

## DISCLOSURES

No conflicts of interest, financial or otherwise, are declared by the authors.

## AUTHOR CONTRIBUTIONS

T.O. and M.M. conceived and designed the research; T.O. and M.M. performed the experiments; T.O. analyzed the data; T.O., R.K., K.I., A.A., S.S., and M.M. interpreted the results of experiments, drafted the manuscript, and approved the final version of the manuscript.
